# Effective Augmentation of Complex Networks

**DOI:** 10.1038/srep25627

**Published:** 2016-05-11

**Authors:** Jinjian Wang, Xinghuo Yu, Lewi Stone

**Affiliations:** 1 School of Engineering, RMIT University, Melbourne, 3000, Australia; 2 School of Sciences, RMIT University, Melbourne, 3000, Australia

## Abstract

Networks science plays an enormous role in many aspects of modern society from distributing electrical power across nations to spreading information and social networking amongst global populations. While modern networks constantly change in size, few studies have sought methods for the difficult task of optimising this growth. Here we study theoretical requirements for augmenting networks by adding source or sink nodes, without requiring additional driver-nodes to accommodate the change i.e., conserving structural controllability. Our “effective augmentation” algorithm takes advantage of clusters intrinsic to the network topology, and permits rapidly and efficient augmentation of a large number of nodes in one time-step. “Effective augmentation” is shown to work successfully on a wide range of model and real networks. The method has numerous applications (e.g. study of biological, social, power and technological networks) and potentially of significant practical and economic value.

Network expansion or augmentation is a ubiquitous feature in our rapidly growing technological society. It manifests in numerous and diverse scientific fields that include indexing and searching of the internet^1^, information dissemination on social networks^2^,^3^, financial and banking networks^4^,^5^, power distribution networks^6^, epidemic outbreaks in human populations^7^, and a vast range of computer applications from virus spread^8^,^9^ to cyber warfare^10^. Expansion often occurs naturally, as in social networks, where new users may join and instantly create new connections. For other networks, increases in demand often naturally lead to growth. For example, higher electricity usage requires the connection of new power stations and loads to the existing power network to balance the extra demand. In biological contexts, where many exciting frontier network projects are now appearing, questions arise as for example, how can one design a “brain on a chip” by engineering neuronal network growth^11^. An immediate and important question, then, is how connections should be added from the existing network so that a certain optimal performance is achieved such as maintaining or enhancing overall controllability. Despite the widespread necessity to augment networks, and the intense scientific interest in network science, very little is known about how one might begin to expand a network subject to controllability constraints. Here we provide the first systematic study that sets out to solve some of these problems.

To address the question, a necessary stepping stone is to understand the controllability of complex networks^12^,^13^,^14^,^15^,^16^,^17^,^18^,^19^. According to control theory^20^,^21^, a linear time-invariant system that is controllable has the capability of being driven from any initial state to any desired final state within finite time. It is well known that controllability can be achieved by modifying the state of a small number of nodes in the network denoted as driver nodes^12^,^22^,^23^. Recent advances in applying control theory to complex networks show that the minimum number of drivers (*N*_*D*_) necessary to yield control over the whole network can be simply determined by the graph theoretic technique of maximum matching^12^,^24^ which is just the maximum set of edges that do not share start or end nodes^25^. A node is defined to be ‘matched’ if one matching edge points to it. *N*_*D*_ equals to number of unmatched nodes. The set of unmatched nodes is referred to as the Minimum Driver node Set (MDS)^12^,^26^. Correspondingly, we denote the Minimum Terminal node Set (MTS) as the set of unmatched nodes in the maximum matching of its transpose network (Supplementary Fig. S1).

We study node augmentation of arbitrary directed networks while insisting that *N*_*D*_ remain unchanged. In the present work we analyse the addition of nodes having only a single edge so that they are either source nodes (having zero in-degree *k*_*in*_ = 0) or sink nodes (having zero out-degree *k*_*out*_ = 0). Figure 1a provides an example of a 57 node network that can be fully controlled with *N*_*D*_ = 11. In Fig. 1b, the network in Fig. 1a is augmented by using our method and adding 10 new nodes (shown in triangles which includes 8 source nodes and 2 sink nodes) while *N*_*D*_ = 11 remains unchanged. However, had these ten nodes been augmented randomly, it is highly unlikely that *N*_*D*_ would remain constant, and conceivably *N*_*D*_ could have increased by as much as ten.

It is always possible to connect a new source node to an existing driver node without changing *N*_*D*_. This is a basic result from structural control theory based on the stem/cactus structure of the network^12^. Thus adding source nodes to the current driver nodes is considered a trivial solution to the augmentation problem, and therefore one that we do not discuss in depth or make use of in this paper. Similarly adding a sink node to a terminal node should be considered as a trivial solution to the augmentation problem. Hence, the main goal of the present paper is encapsulated in the following questions: How can one add source nodes to an existing network other than to the current driver nodes (the trivial solution), while ensuring *N*_*D*_ remains unchanged? Similarly how can one add sink nodes in a non-trivial way under the same constraint? And is it possible to find multiple solutions that can accomplish the same goal?

In general we are interested in determining many possible solutions to the augmentation problem. The main advantage of having multiple solutions to choose from is: with different applications, new nodes might need to be connected to quite different positions in the network in order to take into account spatial constraints. The position may need to be selected from a set of possible solutions based on the modified networks reliability, stability, vulnerability etc. For example, adding ten new nodes (substations, load, and buses) to a power network, in a way that minimises cost, maximises system reliability while ensuring *N*_*D*_ is unchanged. Our methodology finds multiple combinations of nodes other than the trivial existing drivers to connect to. Naturally, we also acknowledge that there is always the option of connecting to the existing drivers (i.e., the trivial solution).

Network augmentation via the addition of edge connections rather than adding nodes has previously been examined from the controllability perspective^27^,^28^,^29^. In these studies, augmentation was implemented by only adding new edges, with the aim of decreasing *N*_*D*_ and therefore enhancing controllability. However, the growth of networks involves not only increasing the number of edges but also the number of nodes. For example, in a supply chain network^30^, new storage warehouses (nodes) may need to be built in order to meet gradually increasing commodity demands. To our knowledge, currently there are no publications that address the augmentation of nodes to a network subject to controllability constraints, as is the goal of our work here.

## Results

### Node classification

**Figure 2:**
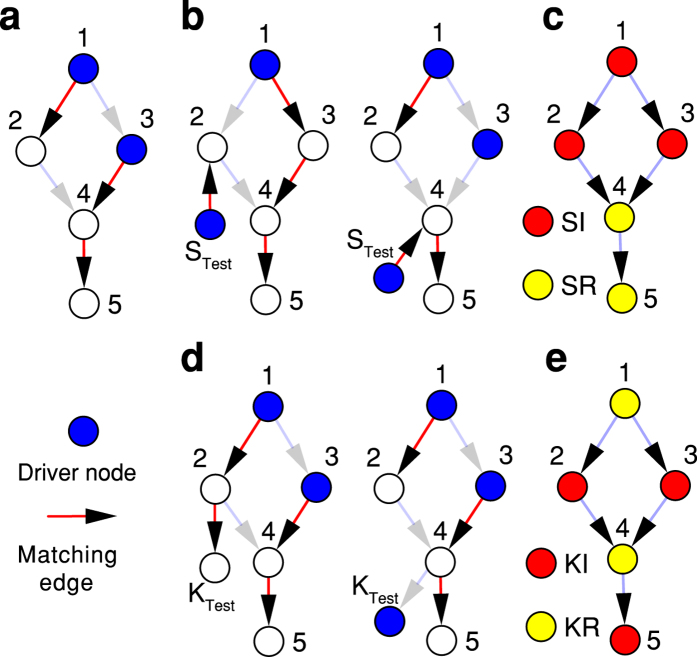
Node Classification of a small network. (**a**) A directed network with five nodes can be controlled via minimum of two driver nodes determined from maximum matching (MDS = {1, 3}). The driver nodes are coloured in blue and the matching edges are marked in red. (**b**) For sources augmentation, nodes are categorized into SI and SR nodes. Node 2 is an SI node since *N*_*D*_ remains unchanged after connecting a new test source node *S*_*Test*_ to it. The same method determines that node 4 is SR. (**c**) SI nodes {1, 2, 3} marked in red and SR nodes {4, 5, 6} coloured in yellow. For sink augmentation, nodes are divided into KI and KR. (**d**) Connecting an independent sink node *K*_*Test*_ to node 2 and 4 respectively, reveals that node 2 is KI (*N*_*D*_ = 2) and node 4 is KR (*N*_*D*_ = 3). The categories are shown in (**e**).

To explore source augmentation, we examine the consequence of connecting a single source test node to any node *x* in an arbitrary directed network G(**A**), where 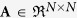 is the state matrix and *N* is total number of nodes. We define the node *x* to be: i) Source Invariant (SI) if *N*_*D*_ does not change, and ii) Source Redundant (SR) if *N*_*D*_ increases. Obviously all driver nodes in a network are SI. Thus, in the example of Fig. 2a, nodes 1, 2, 3 are SI. Adding a source node to any of these nodes leaves *N*_*D*_ unchanged (Fig. 2b). Nodes 4 and 5 are SR (Fig. 2c).

Similarly, for sink augmentation, it is useful to examine what happens when a sink test node is added to any node *x* in the network (Fig. 2d). We define node *x* to be: i) Sink Invariant (KI) if *N*_*D*_ remains unchanged (e.g., node 2, 3, 5 in Fig. 2e); ii) Sink Redundant (KR) if *N*_*D*_ increases. It is easy to prove that source nodes are always SI and sink nodes are KI. Then, we have *N*_*SI*_ + *N*_*SR*_ = *N*, *N*_*KI*_ + *N*_*KR*_ = *N* where *N*_*SI*_, *N*_*SR*_, *N*_*KI*_ and *N*_*KR*_ are the number of nodes of each type. We have proved that a network’s full set of SI nodes is the union of all nodes in every possible MDS. Similarly, full set of KI nodes is the union of all nodes in every possible MTS (Supplementary Note 1).

### Effective source augmentation

According to the definitions of node classification, it is always possible to augment a network by adding a single source node to an SI node, or a single sink node to a KI node without changing *N*_*D*_. However, the method is problematical when there is a need to augment more than one single node in parallel, since the classifications of many nodes in the network change every time a new node is added. In theory, nodes can be augmented serially one after the other but the node classification procedure would need to be performed for every node of the network for each step. When the size of a network is large, the method becomes extremely inefficient.

**Figure 3:**
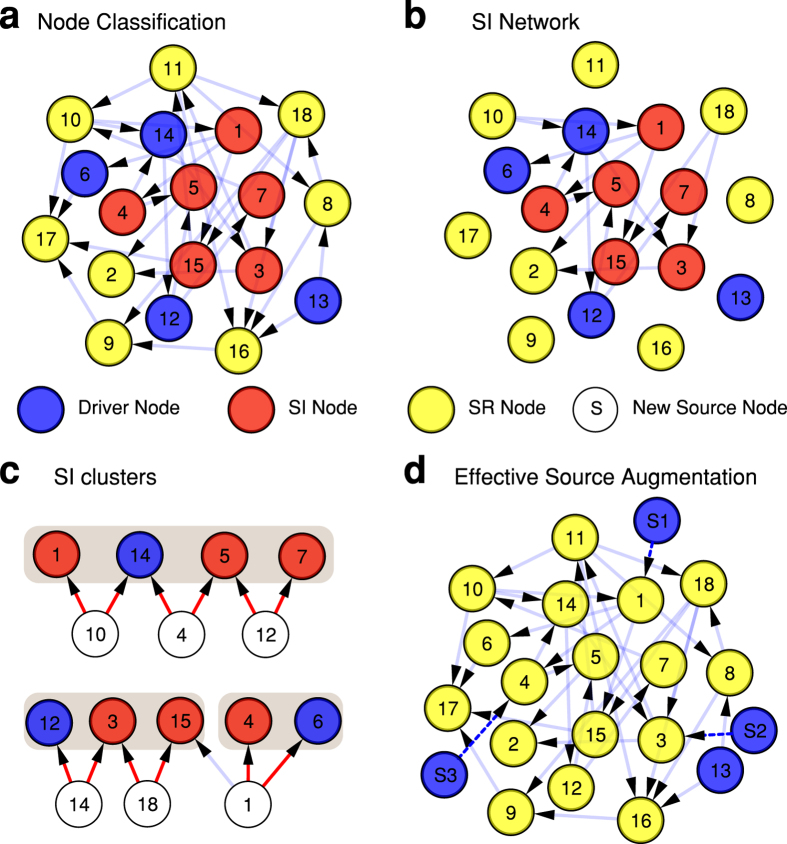
Effective augmentation of a small network. (**a**) A directed network with eighteen nodes can be controlled via the MDS = {6, 12, 13, 14} which is determined by maximum matching and coloured in blue. By applying the node classification algorithm, the nodes are divided into different categories: SI nodes (non-drivers) {1, 3, 4, 5, 7, 15} are red (drivers are always SI), and SR nodes {2, 8, 9, 10, 11, 16, 17, 18} are yellow. (**b**) The SI network is determined by removing all incoming edges to every SR node (yellow) while the outgoing edges remain. This makes the V-motifs easy to identify. (**c**) Multiple V-motifs are identified. They are: 10 → {14, 1}, 4 → {14, 5}, 12 → {5, 7}, 14 → {12, 3}, 18 → {3, 15}. Then, based on the V-motifs, we can identify two SI clusters {1, 5, 7, 14} and {3, 12, 15}. Note that, the structure 1 → {15, 4, 6} is not a V-motif since it involves more than two groups. However, when removing all finalised clusters, 1 → {4, 6} become a V-motif and {4, 6} is a new SI cluster. (**d**) To verify the features of clusters, three independent source nodes are augmented to each cluster, *S*_1_ → 1, *S*_2_ → 3, *S*_3_ → 4. The augmented network remains structurally controllable with the same *N*_*D*_ = 4 but with a different MDS, now (13, *S*_1_, *S*_2_, *S*_3_). The nodes in the clusters have all become SR as expected, and marked yellow, as checked via maximum matching.

However, there is a way around this problem. We find that SI nodes are not isolated but are “correlated” and may be grouped into distinct clusters such that connecting a new node to an SI node affects all others in the cluster in the same way. As such, it is possible to identify a set of clusters in the network, each cluster consisting of a set of correlated SI nodes. The network shown in Fig. 3a contains 10 SI nodes (red and blue) and 8 SR nodes (yellow). The SI nodes are further grouped into three clusters, {14, 1, 5, 7}, {12, 3, 15}, {4, 6} (Fig. 3c shade areas). The unique features of these clusters are that: i) Each cluster contains only one driver node (blue). ii) The number of clusters is exactly the number of sources that can be added to the network in *one* time-step without increasing *N*_*D*_. If there are *k* clusters, it is possible to augment the network with *k* source nodes in parallel. iii) Once a source node is connected to any node in an SI cluster, all nodes in the cluster become SR and they can be ignored from this point in the augmentation process. iv) A newly connected source node replaces the driver node in a cluster, and thereby result in the network having a new MDS. Figure 3d shows that when source nodes (*S*_1_, *S*_2_, *S*_3_) are connected to the three different clusters, all nodes in the clusters change to SR but *N*_*D*_ = 4 remains unchanged, which verifies the properties of clusters. Since all nodes apart from the drivers have become source nodes, from this point no new augmentation is possible.

### Identifying clusters. SI-network

To locate all clusters, it is necessary to first simplify the network by forming the so-called ‘SI-network’, which is easier to work with. The SI-network is obtained by first locating all SR nodes and then removing all of their incoming edges (i.e., setting 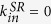). This procedure fragments the original network. The connected network that remains is defined as the SI network, since it contains all SI-nodes, as shown in Fig. 3b. The following discussion concerning clusters only refers to the SI-network.

### V-motif

**Figure 4:**
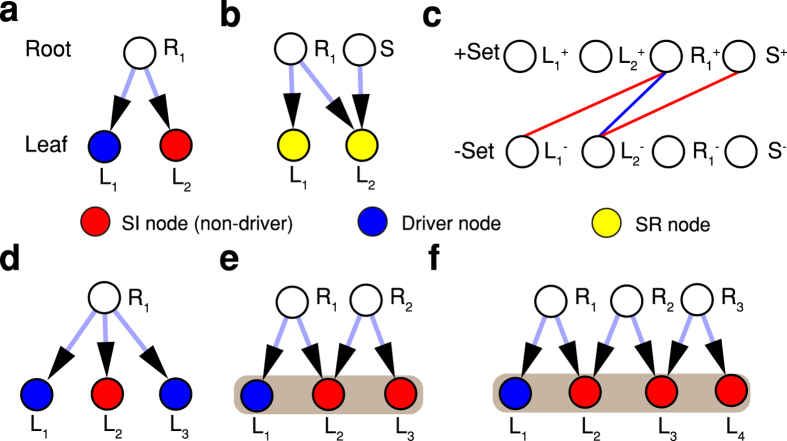
Motifs in SI networks. (**a**–**c**) A demonstration of the simplest SI cluster and its features. (**a**) The prototypical V-motif in source augmentation consists of one root node (SI or SR) pointing to two SI leaves {*L*_1_, *L*_2_}. Nodes *L*_1_, a driver, and *L*_2_ form the simplest SI cluster. For example, when an independent source node ‘S’ connects to *L*_2_ (**b**), both *L*_1_ and *L*_2_ become SR. This can be well explained with maximum bipartite matching of the structure (**c**), where both leaves become always matched. Furthermore, node ‘S’ replace *L*_1_ and becomes the new driver node (become unmatched in the -set). (**d**) A node configuration that has no cluster since it has no V-motif. (**e**) Here nodes *L*_1_, *L*_2_ and *L*_3_ form a cluster being correlated via two intersecting V-motifs. (**f**), similar forms of four-nodes cluster via three intersecting V-motifs.

The simplest and most elementary cluster of SI-nodes possible is referred to as a V-motif and is the basis for clusters of all types. Figure 4a, displays a typical V-motif in the SI-network for the nodes *R*_1_ → {*L*_1_, *L*_2_}, where root-node *R*_1_ points to two and only two leaf nodes *L*_1_ and *L*_2_. The nodes {*L*_1_, *L*_2_} form an elementary SI cluster as long as any one of the nodes is a driver.

This two-node cluster has the fundamental property that its two constituent nodes *L*_1_ and *L*_2_ cannot both be drivers and thus can never participate in the same MDS. The property can be inferred from a simple maximum matching of the V-motif. (We see that if an independent source node ‘S’ is augmented to *L*_2_, then every node of the SI cluster becomes matched (Fig. 4c) and thus all nodes in the cluster {*L*_1_, *L*_2_} change to SR nodes (Fig. 4b). The new node ‘S’ become unmatched and replaces *L*_1_ as the new driver node. Hence *N*_*D*_ remains conserved.) Moreover for other isolated motifs of the form, say, *R*1 → {*L*_1_, *L*_2_, *L*_3_} (Fig. 4d) which is not a V-motif, maximum matching shows that the nodes *L*_1_, *L*_2_, *L*_3_ lack the above fundamental property.

Building on the example of the V-motifs, it is possible to obtain three-node clusters and multiple-node clusters etc., each of which has the same fundamental property: there can only be one driver node within any cluster. Figure 4e gives an example of a three-node cluster (grey region) obtained from two intersecting V-motifs. Here root-node *R*_1_ points to leaves *L*_1_ and *L*_2_ while root-node *R*_2_ points to leaves *L*_2_ and *L*_3_. The set of SI nodes *L*_1_, *L*_2_ and *L*_3_ form a cluster if any one of these nodes is a driver. Figure 4f gives an example of a four-node cluster obtained from three intersecting V-motifs. For networks with different types and varying degree distributions, V-motifs can become entangled in the SI network but may nevertheless be identified by these methods (Supplementary Fig. S2 and Supplementary Note 2).

### Effective sink augmentation

Similarly, the cluster concept also holds for sink augmentation. The ‘KI network’ (Supplementary Fig. S3) can be formed by removing outgoing edges of KR nodes (i.e., setting 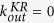) instead of incoming edges. Now, the V-motif (Supplementary Figs S3 and S4) in the KI network is the same as that we have considered, but directionally reversed. Multiple KI nodes are collected in the same KI cluster if: i) KI nodes are correlated via V-motifs in the KI network; ii) there is one KI node that belongs to the MTS.

A network can be effectively augmented up to the number of KI cluster (*N*_*KIC*_) new sink nodes. For each cluster it is only possible to connect a single sink node without changing *N*_*D*_ (Supplementary Fig. S3) and after augmentation, all nodes in the cluster become KR. Furthermore, we find that sink augmentation has no effect on the current MDS (Supplementary Note 3). Thus sources and sinks augmentation can be implemented in parallel without interference. These features make network augmentation very efficient and flexible. We have developed an algorithm to search all possible V-motifs in both SI and KI networks, and then determine all possible SI and KI clusters in linear time (see Methods).

### Effective augmentation in synthetic networks

We have illustrated the importance of clusters in network augmentation. As the minimum number of new source (or sink) nodes that can be augmented by effective augmentation method depends on the numbers of SI (or KI) clusters existing in the network, there is a need to explore the latter empirically. Denote the number of SI clusters in a network as *N*_*SIC*_ and the number of KI clusters as *N*_*KIC*_. Base on their properties, we understand that SI clusters never contain source nodes and each cluster has exactly one non-source driver node. Thus for an arbitrary network G(**A**) the upper limit of *N*_*SIC*_ must be *N*_*MAS*_ = *N*_*D*_ − *S*, where *N*_*MAS*_ is the maximum number of augmentable source nodes, and *S* is the number of source nodes in **A**. Similarly, *N*_*KIC*_ is limited with *N*_*MAK*_ = *N*_*D*_ − *K*, where *K* is the number of sink nodes in **A**.

**Figure 5:**
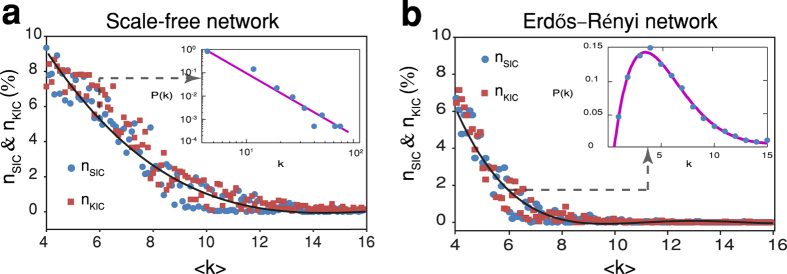
Effective augmentation of synthetic networks. Distribution of the minimum fraction of new source nodes (*n*_*SIC*_) and sink nodes (*n*_*KIC*_) that can be effectively augmented in scale-free networks (identical degree exponents *γ*_*in*_ = *γ*_*out*_ = 3) and Erdös-Rényi networks respectively. (**a**) *n*_*SIC*_ and *n*_*KIC*_ versus 〈*k*〉 are shown in blue dots and red squares respectively. These networks have a power law degree distribution. Insert figure shows the degree distribution of the network with 〈*k*〉 = 6. The solid line shows the curve fitting of the presented data using non-linear polynomial fitting functions. (**b**) *n*_*KIC*_ and *n*_*KIC*_ in Erdös-Rényi networks with varying 〈*k*〉. The degree distribution of ER graphs converges to a Poisson distribution (insert).

We first study scale-free networks^31^,^32^ (Supplementary Note 4) of different average degree but having identical input and output degree exponents (*γ*_*in*_ = *γ*_*out*_ = 3). Without loss of generality, we consider here networks with no disconnected nodes. For the purposes of exploring the cluster distributions over different network sizes, we normalise the parameters based on the network’s total number of nodes, N, respectively, i.e., (*n*_*SIC*_, *n*_*KIC*_, *n*_*D*_) = (*N*_*SIC*_, *N*_*KIC*_, *N*_*D*_)/*N*. Figure 5a demonstrates that *n*_*SIC*_ (blue dots) and *n*_*KIC*_ (red squares) follow the same trend: both decrease as the mean degree of the network increases. For networks with low average degree, e.g., 〈*k*〉 = 4 the minimum fraction of new nodes that can be augmented in parallel is approximately 9% of the total number of nodes (*n*_*SIC*_ = 9%). For networks with high mean degree, augmentation is limited independent of the method. For example, in a fully connected network with *N*_*D*_ = 1, only one new source node can be augmented and *n*_*SIC*_ = 0 since no V-motifs exist.

To investigate the capability of the method in capturing a high proportion of augmenting solutions, the number of clusters *N*_*SIC*_ (blue dots) and *N*_*MAS*_ (yellow triangles; the upper limit of augmentation) are plotted as a function of average degree 〈*k*〉 in Supplementary Fig. S5a. At 〈*k*〉 = 4, approximate 65% (*N*_*SIC*_/*N*_*MAS*_) of the total possible source nodes *N*_*MAS*_ can be augmented simultaneously via our cluster based method in SF network. Again, Fig. S5a shows that the higher the mean degree of the network, the less the number of new nodes that can be augmented non-trivially.

To explore *n*_*SIC*_ and *n*_*KIC*_ over different network types, we further investigate the parameters in Erdös-Rényi networks^33^ (Fig. 5b, Fig. S5b, Supplementary Note 4). We find that ER networks are less augmentable (*N*_*SIC*_/*N*_*MAS*_ ≈ 90% at 〈*k*〉 = 4) compared to SF networks (65% at same 〈*k*〉). This is due to the fact that in general ER networks require less driver nodes to achieve full control when compared to SF networks having identical network size and mean degree^12^. Intriguingly, we find that in ER networks, the decrease of *n*_*SIC*_ and *n*_*KIC*_ as a function of 〈*k*〉 are sharper compared with SF networks. When the average degree exceeds a critical value *k*_*c*_ ≈ 8, the networks become almost non-augmentable. This is because the maximum number of source nodes that can be augmented, *N*_*MAS*_, reaches approximately zero at *k*_*c*_ ≈ 8 (Supplementary Fig. S5b). On the other hand, *N*_*MAS*_ approaches zero when *k*_*c*_ ≈ 13 in SF-networks. Thus SF networks can be augmented within broader average degree regions than ER networks. Furthermore, we find the effective augmentation method can be applied more efficiently in ER networks as compared to SF networks. This can be observed clearly in Supplementary Fig. S5 which shows that the difference between *N*_*SIC*_ and *N*_*MAS*_ for ER networks is much smaller than their difference in SF networks (compare Fig. S5a,S5b).

### Effective augmentation in real networks

**Figure 6:**
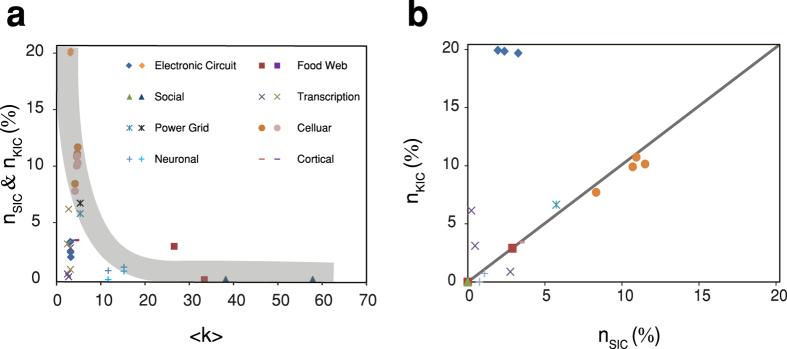
Effective augmentation of real networks. (**a**) The fraction of clusters versus mean degree for real networks. The left and right symbols associated with each network type in inset figure represent *n*_*SIC*_ and *n*_*KIC*_ respectively. Fraction of clusters follow decreasing trend over 〈*k*〉 (highlighted bands). (**b**) For same real networks, *n*_*SIC*_ versus *n*_*KIC*_ is plotted.

To demonstrate the feasibility of the augmentation algorithm, we apply the tools developed above to several real networks from a variety of different applications (Table 1, Supplementary Tables S1 and S2). Figure 6a shows the fraction of clusters (both SI and KI clusters) determined from real networks versus mean degree. Overall, both *n*_*SIC*_ and *n*_*KIC*_ decrease as 〈*k*〉 increases which is in agreement with what was found from simulations of ER and scale-free networks. For example, networks as the cellular, electronic circuit and power grid with relatively low average degree have many more clusters and are thus much more augmentable than networks having large average degree, such as social networks.

It is also important to note that some networks exhibit different proportions of SI and KI clusters. We find that electronic networks can be readily augmented with sink nodes (*n*_*SIC*_ ≈ 20%) but are limited with source nodes (*n*_*KIC*_ ≈ 3%). To further investigate this property, we plot *n*_*SIC*_ versus *n*_*KIC*_ in Fig. 6b and *n*_*SIC*_, *n*_*KIC*_ versus *n*_*D*_ in Supplementary Fig. S6. We find that some networks, such as cellular, food web, power grid, social networks have approximately the same fraction of SI and KI clusters, while other networks, like electronic circuit (more augmentable with sink nodes than source nodes) and transcription networks (Yeast networks are more augmentable with source nodes than sink nodes) are away from the diagonal line. This may be caused by different in- and out- degree distributions and degree asymmetries^18^,^34^,^35^. Furthermore, large numbers of SI clusters and KI clusters are found in cellular networks, power grid and cortical networks indicating that these sorts of networks can be augmented efficiently with the clustering method used in our effective augmentation. Finally, we also observed that networks from the same categories have similar *n*_*SIC*_ and *n*_*KIC*_, which means they have similar augmentation properties.

## Discussion

The cluster method proposed here allows us to generate a relatively large number of workable solutions in one time-step. For our method, the number of ways of adding *m* new nodes to the network non-trivially with *n* clusters (assuming each cluster has *k* nodes) is 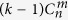 where *m* ≤ *n*, which is usually quite considerable. Furthermore, the solutions allows connecting multiple source and sink nodes in parallel simultaneously without conflict, which increases the effectiveness of the method. It might seem a limitation that the newly connected source and sink nodes are required to be of degree one (i.e., have one edge). However, by taking advantage of the clusters present, it is a straightforward extension of the method to augment new nodes having higher degree. Each new edge can be attached to a different cluster.

Our approach is based on the presence of V-motifs in both the SI and KI network. When the method is applied to random networks with varying average degree, we find that the ability to augment a network is inversely proportional to its average degree. This is attributed to the fact that the network clusters reduce in number as network connectivity and average degree increase. Networks having large average degree tend to have few clusters, and thus are hard to be augmented in practice, as was found for social networks. For real networks, networks from the same categories tend to have similar fractions of clusters. In summary, we have formulated a successful and efficient procedures for augmenting arbitrary directed networks while keeping the minimum number of driver nodes required to fully control the network.

## Methods

### Identifying minimum driver node set and minimum terminal set

The MDS and MTS of a directed network G(*V*, *E*) can be identified by the following steps. 1) Bipartite representation (Supplementary Fig. S1): divide node set *V* into two disjoint node sets + and −, such that any directed edge (*V*_*i*_ , *V*_*j*_) ∈ E can be represented as (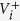, 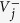) in the bipartite graph G(*V*^+^ ∪ *V*^−^, E). 2) Maximum bipartite matching: determine the maximum matching of the bipartite graph with Hopcroft-Karp algorithm^25^,^36^. MDS and MTS are unmatched nodes in the − set and + set respectively. The number of computational steps needed to find one maximum matching can be as small as *O*(*N*^0.5^*L*) with the Hopcroft-Karp algorithm, where *N* and *L* represent the number of nodes and edges in the network respectively.

### Identifying SI and KI clusters

Nodes in a directed network G(*V*, *E*) should be classified into two sets: SI and SR. To expedite finding SI clusters it is helpful to first form the corresponding SI network defined as the remaining connected sub-graphs found after removing all incoming edges to all SR nodes. The identification of SI clusters is then achieved through a sequential process of searching for V-motifs. The search begins with low out-degree nodes and ends with nodes having highest out-degree. The process makes use of cluster merging. Two clusters (sets of nodes) can be merged into one if: i) the two clusters appear as two distinct leaves of a V-motif; ii) the two clusters contain one or more common nodes. The detailed procedure is as follows:

1. Search the SI network for all nodes having out-degree equal two (*k*_*out*_ = 2). If there are no nodes with *k*_*out*_ = 2 in the SI network there will be no clusters. Each identified node is the root node of a prototypical V-motif, and the two nodes connected to the root are leaves. Being part of the SI network, they must both be SI. From here on, treat both of these SI nodes as a merged cluster. Multiple clusters are merged if they meet the above merging conditions i) or ii). For example, from the V-motifs in Fig. 3c, the sets of SI nodes {14, 1}, {1, 5} and {5, 7} are considered as three clusters (merge condition i). The clusters can be further merged together as {1, 5, 7, 14} (merge condition ii).
2. Search for all nodes with out-degree equal to three. First, each of these root nodes has three leaves needs to be merged into clusters where possible. A V-motif is formed if each root node points to two and only two distinct clusters, e.g., In Supplementary Fig. S2a, *R*_2_ → {*L*_1_, *L*_2_, *L*_3_} is a V-motif, since *L*_1_ and *L*_2_ belongs to the same cluster. Finally, all clusters identified should be merged where possible before move to the next step.
3. Repeat procedure (2) iteratively on nodes after increasing *k*_*out*_ by one each step. The process terminates when either no V-motifs can be found, or in the worst case terminated on nodes with highest possible *k*_*out*_ value. Finally, the desired SI clusters are those clusters which contain more than one nodes, one of which must be a driver node. Distinct SI clusters do not share common nodes.
4. Due to the complexity of the network, the first implementation of the above procedure will not identify all possible SI clusters in the network. However, the remaining SI clusters can by identified by eliminating the existing SI clusters from the SI network (delete all nodes in the SI clusters along with their edges) and repeat procedures (1)–(3) until no further V-motifs can be found. This will eventually reveal all possible SI clusters.

The identification of KI clusters follows the same procedures except that it is necessary to begin with the KI network which is determined by removing incoming edges to KR nodes. A standard V-motif in the KI network is defined as one leaf node pointed to by two KI root nodes (Supplementary Fig. S4). Similarly, KI cluster identification follows the same procedures except searching begins with low in-degree (*k*_*in*_ = 2) nodes. The conditions of two clusters merge together in sources augmentation holds for sinks augmentation as well. SI (or KI) clusters determination is based on the degree of individual nodes in the SI (or KI) network. Thus, the complexity is proportional to number of nodes and edges in the SI (or KI) network. Therefore, the complexity of the algorithm is linearly proportional to *N* and *L* in the network.

## Additional Information

**How to cite this article**: Wang, J. *et al.* Effective Augmentation of Complex Networks. *Sci. Rep.* **6**, 25627; doi: 10.1038/srep25627 (2016).

## Supplementary Material

Supplementary Information

## Figures and Tables

**Figure 1:**
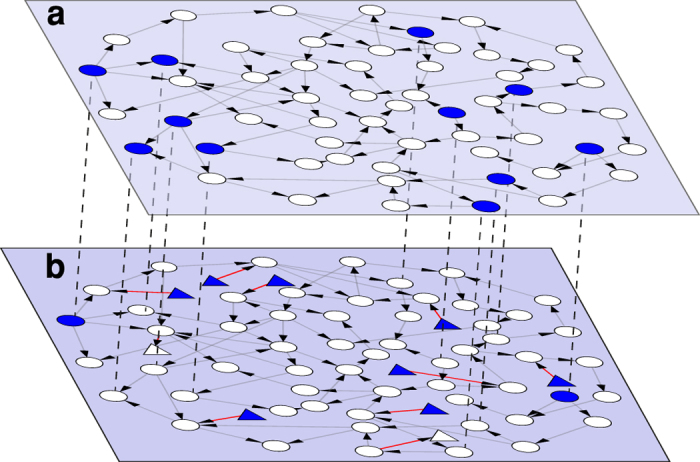
Example of 57 nodes network augmentation. (**a**) A directed network with fifty-seven nodes can be controlled via a minimum of eleven driver nodes (*N*_*D*_ = 11) coloured in blue. (**b**) The network in (**a**) is now augmented by eight independent source nodes and two sink nodes, and are displayed as triangles. The new network can still be fully controlled via *N*_*D*_ = 11 drivers.

**Table 1:** The properties of the real network analysed.

Type	Name	N	L	*n*_*D*_	*n*_*SIC*_	*n*_*KIC*_
ElectronicCircuits	s208	122	189	0.24	3.28	19.67
	s420	252	399	0.23	2.38	19.84
	s838	512	819	0.23	1.95	19.92
Food Web	Everglades	69	916	0.30	2.90	2.90
	Baywet	128	2137	0.23	0	0
	Gramdry	69	915	0.30	2.90	2.90
Social	Cons-frequency-rev	46	879	0.04	0	0
	Manuf-frequency-rev	77	2228	0.01	0	0
Transcription	Ecoli	419	519	0.75	0.48	3.10
	Yeast	688	1079	0.82	2.76	0.87
	ColiInterFullVec	424	577	0.73	0.24	6.13
Power Grid	Dallas	4941	13188	0.12	5.75	6.64
Cortical	Macaque cortical	1168	2486	0.04	3.42	3.42
Neuronal	C. elegans-1	131	764	0.09	0.76	0
	C. elegans-2	277	2105	0.12	1.08	0.72
Cellular	AA	1485	3400	0.29	10.71	9.90
	BB	804	1674	0.27	8.33	7.71
	EF	1407	3290	0.31	10.95	10.73
	PA	2554	6080	0.32	11.51	10.14

For each network, we show its types, name, number of nodes (N), edges (L), fraction of driver nodes (*n*_*D*_), minimum proportion of new source and sink nodes can be augmented in parallel (*n*_*SIC*_ and *n*_*KIC*_). Note that *n*_*SIC*_ and *n*_*KIC*_ are shown in percentage.
