# Supplementary material for: Effective Augmentation of Complex Networks
Source: Sci Rep. 2016 May 11;6:25627. doi: 10.1038/srep25627 (PMC4863250; doi:10.1038/srep25627)
Supplement: Supplementary Information [file srep25627-s1.pdf]

Supplementary Information
Effective Augmentation of Complex Networks

Jinjian Wang[1], Xinghuo Yu *[2], and Lewi Stone[3]

[1]School of Engineering, RMIT University, Melbourne, 3000, Australia.
[2]School of Engineering, RMIT University, Melbourne, 3000, Australia.
[3]School of Sciences, RMIT University, Melbourne, 3000, Australia.

*x.yu@rmit.edu.au

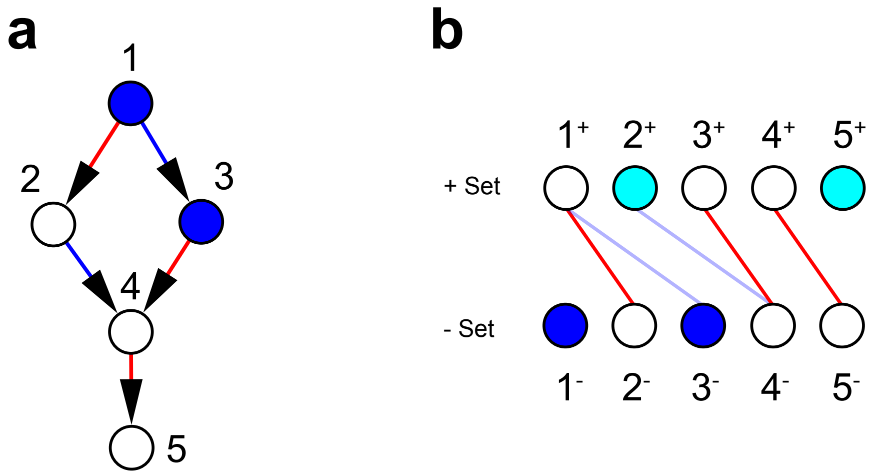

Supplementary Figure S1: **Maximum matching of digraph and its bipartite representation.** (**a**) An example of a five node directed network controlled via the MDS = {1,3}. Red arrows are denoted as matching edges. (**b**) Bipartite representation of the network (a). In the bipartite graph, nodes are converted into two disjoint sets of nodes: + set and - set. Any directed edge from node i to node j in **a** corresponds to a connecting between i^+ and j^-. The MDS is comprised of the unmatched nodes (blue) in the - set, here {1,3}, and the MTS is comprised of the unmatched nodes (cyan) in + set which in this case is {2,5}. The MDS and MTS contain the same number of nodes. Any single network may have multiple maximum matching solutions – there is no unique solution in general. This implies that the network may be controlled by multiple MDSs. Only one of the possible MDSs is shown in this example.

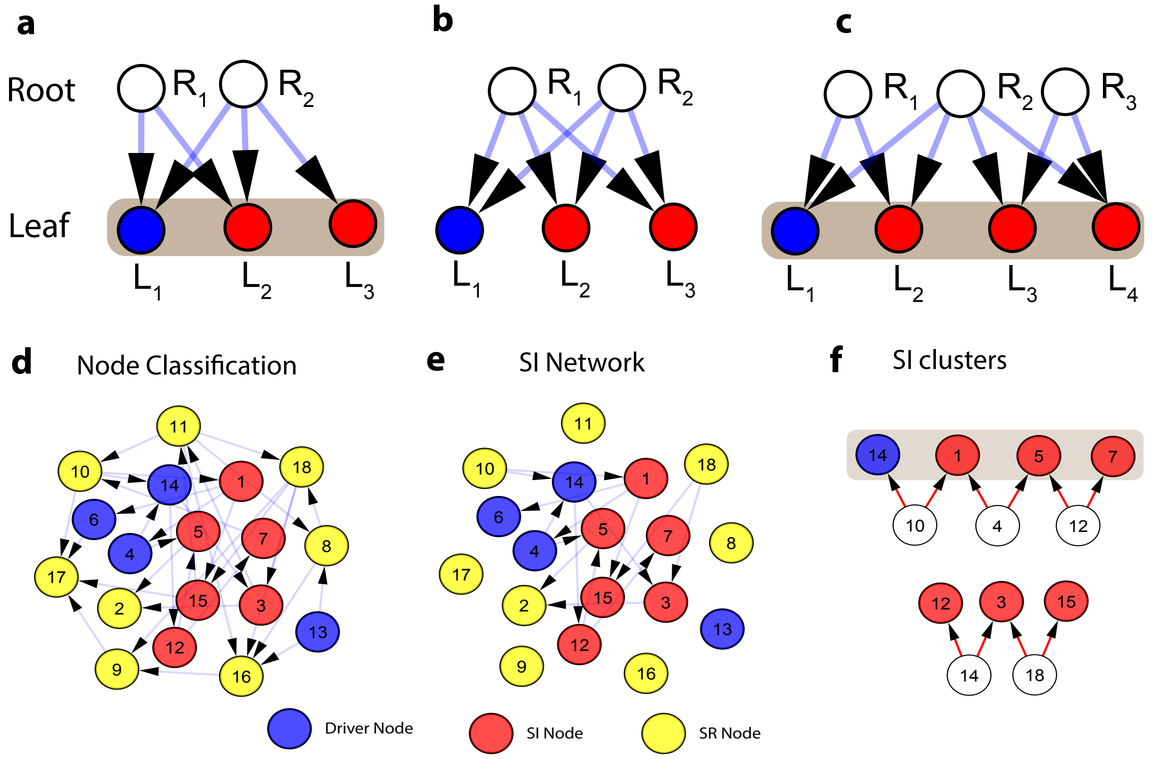

Supplementary Figure S2: **V-motifs in SI networks** (**a**) A more complex three-node cluster, where $R_1 \rightarrow \{L_1, L_2\}$ is a V-motif but $R_2 \rightarrow \{L_1, L_2, L_3\}$ is not. $\{L_1, L_2, L_3\}$ forms a cluster which is shown by the grey shading. (**b**) Shows a similar configuration to a, for which there are no clusters of any type. (**c**) An example of a complex four-node cluster (Supplementary Note 2). (**d**-**f**) An example of SI cluster determination in a complex network with MDS $= \{4, 6, 13, 14\}$. (**d**) Node Classification: determine the nodes into two main types: SR nodes and SI nodes, where driver nodes are always SI. (**e**) SI Network: determine SI network by removing incoming edges to SR nodes. (**f**) SI Cluster: determining the SI clusters by searching for V-motifs in SI network. Only $\{14, 1, 5, 7\}$ is a cluster since it contains a driver.

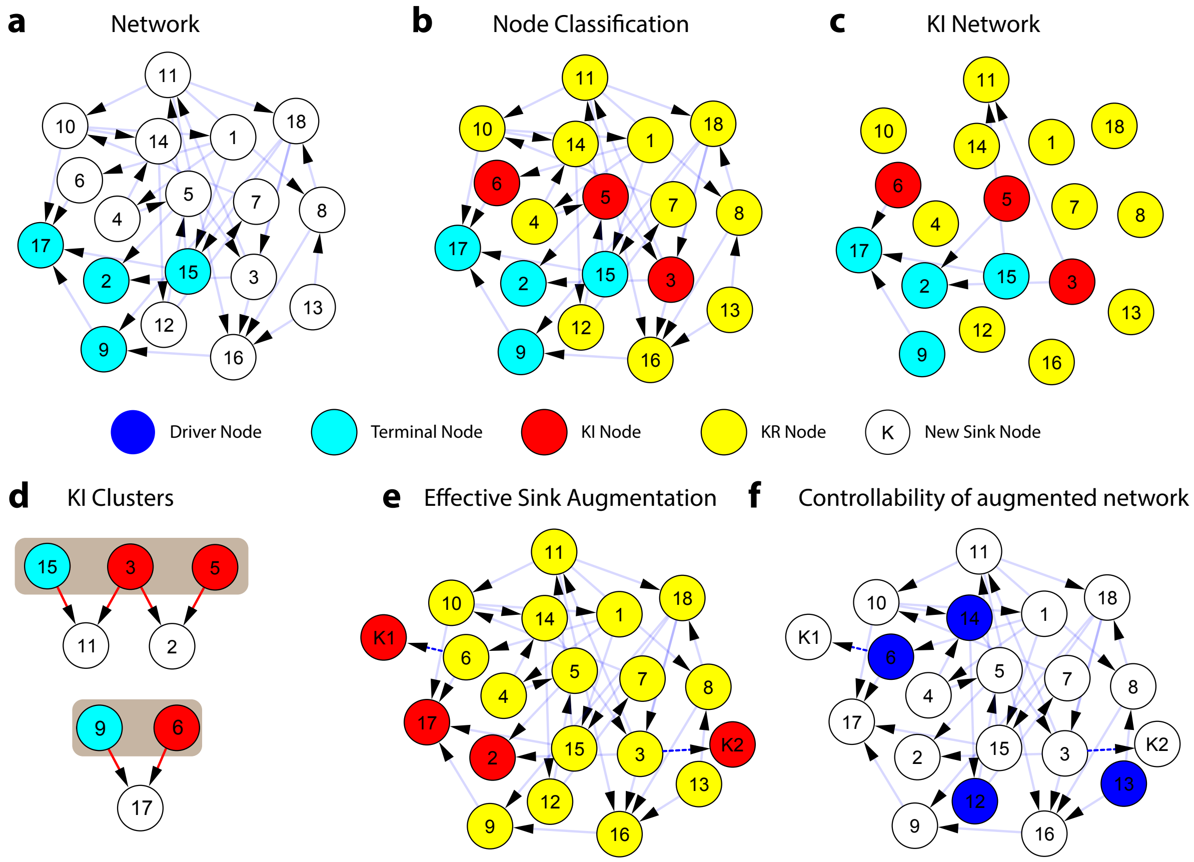

Supplementary Figure S3: **Effective sink augmentation of a small network.** This should be compared with effective source augmentation in Fig.3 main text. (**a**) A directed network with eighteen nodes contains MTS={2,9,15,17} which is determined by maximum matching (Supplementary Figure S1). (**b**) By applying our node classification algorithm, we divide the nodes into different categories: KI nodes (non-terminal nodes) {3,5,6} are red and KR nodes {1,4,7,8,10,11,12,13,14,16,18} are yellow. Terminal nodes are shown in cyan. (**c**) The 'KI network' is determined by removing all outgoing edges from every KR node (yellow) while the incoming edges remain. (**d**) Multiple V-structures are identified in **c**. They are: $\{15, 3\} \rightarrow 11$, $\{3, 5\} \rightarrow 2$, $\{9, 6\} \rightarrow 17$. Then, based on the V-motifs, we can identify two KI clusters {15,3,5} and {9,6}. (**e**) To verify the features of clusters, two independent sink nodes are augmented to each cluster, $6 \rightarrow K_1$, $3 \rightarrow K_2$. By classifying nodes in this new network we find every node in the cluster become KR and the newly connected nodes K_1, K_2 become new terminal nodes while the total number of terminal nodes remain four. (**f**) The augmented network is still structurally controllable with the same MDS as shown in Fig.3 in the manuscript which implies that source augmentation and sink augmentation can be implemented in parallel without conflict.

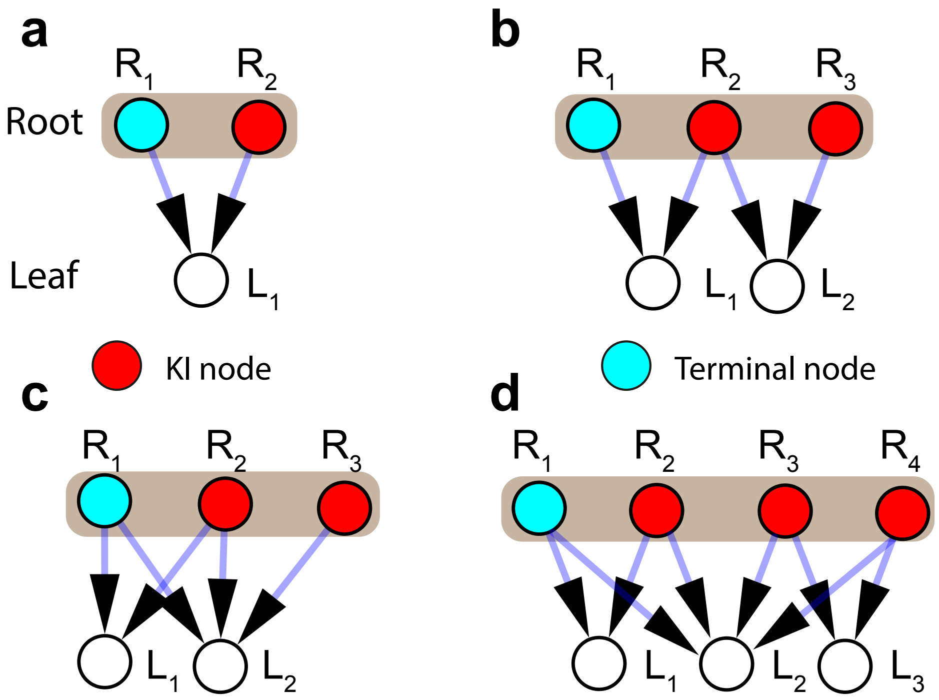

Supplementary Figure S4: **Determining KI clusters via V-motifs in KI networks** (**a-d**) Fragmented sub-graphs in KI networks. The KI nodes are correlated as roots in a 'V-motif' or in multiple 'V-motifs' that lie in the KI network. (**a**) A prototypical V-motif for sink augmentation which is comprised of one leaf node and is pointed to by two root nodes (KI nodes). The two root nodes (R_1, R_2) are considered as a KI cluster since: i) R_1 is a terminal node; ii) two KI nodes are correlated via a V-motif and no other KI nodes are correlated to this terminal node. (**b**) R_1, R_2, R_3 are connected via two serial V-motifs and therefore, $\{R_1, R_2, R_3\}$ is a KI cluster. (**c-d**) More complicated forms of V-motifs. (**c**) $\{R_1, R_2\} \rightarrow L_1$ is a V-motif, thus, $C_1 = \{R_1, R_2\}$ can be considered as a composite node. Then, we find a new V-motif forms as $\{C_1, R_3\} \rightarrow L_2$. Since no other KI nodes are involved, thus, $\{R_1, R_2, R_3\}$ is a KI cluster. (**d**) Using the same concept, we find $\{R_1, R_2, R_3, R_4\}$ is a KI cluster.

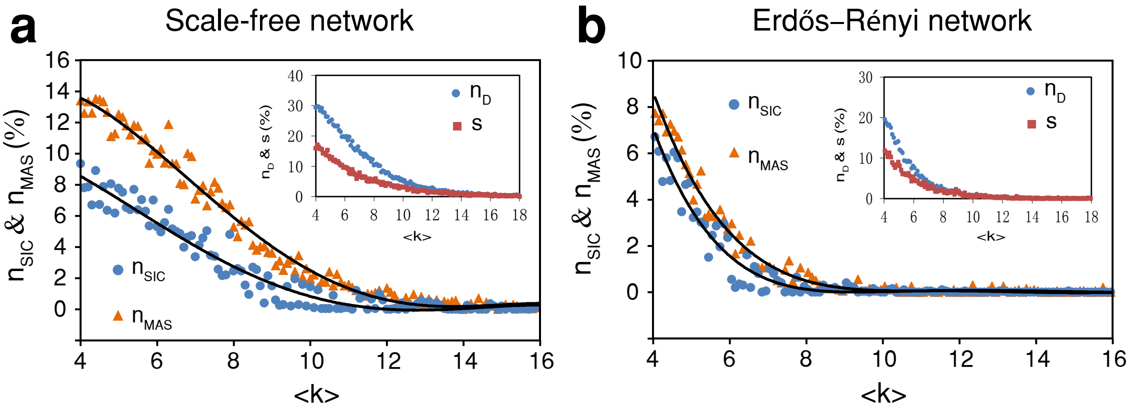

Supplementary Figure S5: **The relationship between nSIC and nMAS in synthetic networks.** (**a**) $n_{SIC} = N_{SIC}/N$ and $n_{MAS}= N_{MAS}/N$ versus $\langle k \rangle$ in scale-free networks are shown in blue dots and yellow triangles respectively, where, N is total number of nodes. $n_D = N_D/N$ and $s = S/N$ versus $\langle k \rangle$ are shown in insert figure. (**b**) n_{SIC} and n_{MAS} in Erdős-Rényi networks is demonstrated.

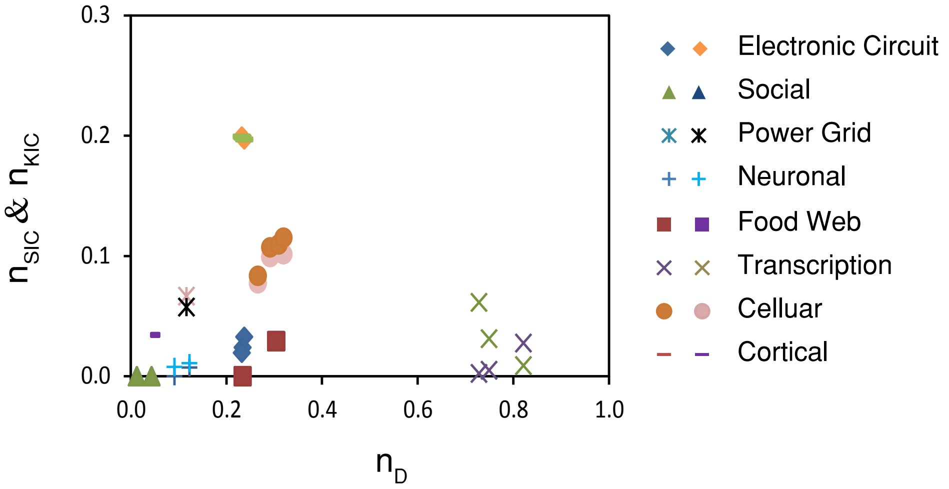

Supplementary Figure S6: **Fraction of SI cluster and KI cluster versus fraction of driver nodes in real networks.** The left and right symbols associated with each network type in figure legend represent n_{SIC} and n_{KIC} respectively.

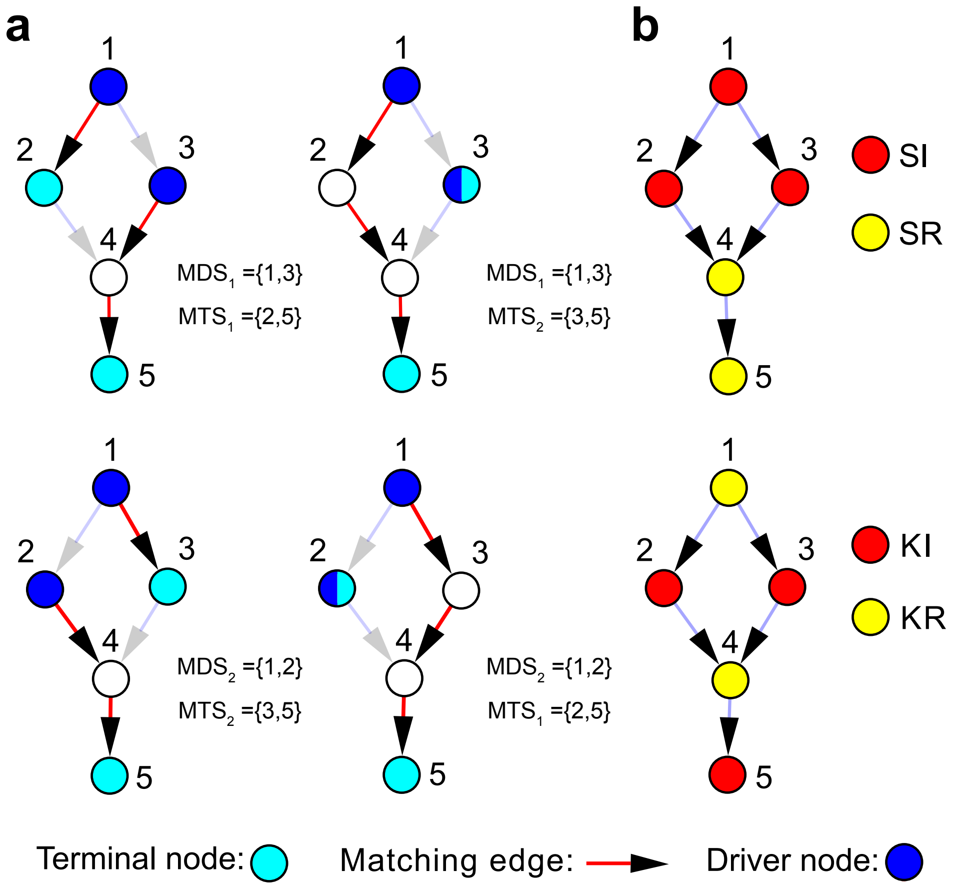

Supplementary Figure S7: **Correlations between MDSs and MTSs.** (**a**) A directed network with five nodes can be controlled via four different maximum matching solutions. Each figure contains two matching paths and each one begins with an driver node (blue) and ends with a terminal node (cyan). Matching edges are coloured in red. (**b**) SI nodes are union of all possible MDSs: $SI = \{1,3\} \cup \{1,2\} = \{1,2,3\}$ as shown in red nodes in top plot. Similarly, KI=$\{2,3,5\}$ is determined from the combination of all MTSs as shown in bottom plot.

Type	Name	Description
Electronic Circuits	s208 [1]	Network parsed from the ISCAS89 benchmark data set
	s420 [1]	Same as above
	s838 [1]	Same as above
Food Web	Everglades [2]	Everglades Graminoid Marshes
	Baywet [3]	Florida Bay, Wet Season
	Gramdry [3]	Everglades Graminoids, Dry Season
Social	Cons-frequency-rev [4]	A consulting company intra-organisational network
	Manuf-frequency-rev [4]	A manufacturing company intra-organisational network
Transcription	Ecoli [5]	Transcriptional regulation network for E.coli
	Yeast [6]	Transcriptional regulation network for Yeast
	ColiInterFullVec [7]	Transcriptional regulation network of Escherichia coli
Power Grid	Dallas [8]	High-voltage power grid network in Dallas
Cortical	Macaque cortical [9]	Macaque cortical connectivity
Neuronal	C. elegans-1 [10]	C. elegans local network of 131 frontal neurons
	C. elegans-2 [11]	C. elegans global network of 277 neurons
Cellular	AA [12]	Cellular network of Aquifex Aeolicus
	BB [12]	Cellular network of Borrelia Burgdorferi
	EF [12]	Cellular network of Enterococcus Faecalis
	PA [12]	Cellular network of Pseudomonas Aeruginosa

Supplementary Table S1: For each network, we show its type, name, reference, and brief description.

**Type**	**Name**	**N**	**L**	$\mathbf{n_D}$	$\mathbf{n_{SIC}}$	$\mathbf{n_{KIC}}$
Electronic Circuits	s208	122	189	0.24	3.28	19.67
	s420	252	399	0.23	2.38	19.84
	s838	512	819	0.23	1.95	19.92
Food Web	Everglades	69	916	0.30	2.90	2.90
	Baywet	128	2137	0.23	0	0
	Gramdry	69	915	0.30	2.90	2.90
Social	Cons-frequency-rev	46	879	0.04	0	0
	Manuf-frequency-rev	77	2228	0.01	0	0
Transcription	Ecoli	419	519	0.75	0.48	3.10
	Yeast	688	1079	0.82	2.76	0.87
	ColiInterFullVec	424	577	0.73	0.24	6.13
Power Grid	Dallas	4941	13188	0.12	5.75	6.64
Cortical	Macaque cortical	1168	2486	0.04	3.42	3.42
Neuronal	C. elegans-1	131	764	0.09	0.76	0
	C. elegans-2	277	2105	0.12	1.08	0.72
Cellular	AA	1485	3400	0.29	10.71	9.90
	BB	804	1674	0.27	8.33	7.71
	EF	1407	3290	0.31	10.95	10.73
	PA	2554	6080	0.32	11.51	10.14

Supplementary Table S2: For each network, we show its types, name, number of nodes (N), edges (L), fraction of driver nodes (n_D), minimum proportion of new source and sink nodes can be augmented in parallel (n_{SIC} and n_{KIC}), Note that n_{SIC} and n_{KIC} are shown in percentage.

Supplementary Note 1: The set of SI and KI nodes is the union of all MDSs and MTSs respectively

Theorem 1: The set of SI nodes of a arbitrary directed network G(A) is the union of all nodes in every possible MDS.

Theorem 1 is equivalent to the following two statements: i) Any node V_i in G(A) is an SI node if it is a member of at least one possible MDS. ii) V_i is SR if it is not a member of any MDS.

Proof: Statement i) If V_i belongs to at least one MDS, then there exists at least one maximum matching solution such that V_i is unmatched. Connecting a new test source node to an unmatched node will not affect the total number of unmatched nodes (i.e., drivers). Thus, according to node classification, V_i is an SI node.

Proof: Statement ii) If V_i is not a member of any MDS, then it never needs to be controlled. Connect a test source node V_S to V_i and assume the new augmented network is still structurally controllable with the same N_D. Since source node V_S has no incoming edges, it is always needs to be controlled. Hence, remove V_S from the network, the remaining network (G(A)) should be fully controlled with $N_D - 1$ driver nodes. This leads to a contradiction. Then, our assumption fails, and the number of drivers N_D required to fully control the new network increases when the test node is added to V_i. Hence, V_i is an SR node.

Theorem 2: The set of KI nodes of a directed network G(V,E) is the union of all nodes in every possible MTS.

Proof: The concepts introduced in *Theorem 1* hold for *Theorem 2*. Denote G(A^T) as a directed network with transposed state matrix A^T. Then, the MTS of G(A) is the MDS of G(A^T). Hence, Theorem 2 is equivalent to proving theorem 1 with the new network G(A^T).

Supplementary Note 2: Determining clusters from complex V-motifs

In the main text Fig. 4, we explained how to locate clusters generated from simple intersecting V-motifs. Here we examine more complex cases that can arise. For example, in Supplementary Figure S2a, the root node $R_1 \rightarrow \{L_1, L_2\}$ is a V-motif but $R_2 \rightarrow \{L_1, L_2, L_3\}$ is not. From studying maximal matching solutions, a simple rule emerges that allows us to accommodate such special cases. In this case we treat L_1 and L_2 as a merged composite node $C_1 = \{L_1, L_2\}$. Then the structure $R_2 \rightarrow \{L_1, L_2, L_3\}$ can be collapsed to $R_2 \rightarrow \{C_1, L_3\}$, which should be viewed as a V-motif. Thus $\{L_1, L_2, L_3\}$ forms a cluster. Maximum matching shows this three node set has the same fundamental property of clusters. Figure S2b shows a similar configuration to Figure S2a, for which there are no clusters of any type. This is because R_1 and R_2 are not root nodes of any V-motif. With the same procedure, in Figure S2c, we identify the V-motif $R1 \rightarrow \{L_1, L_2\}$. Nodes L_1 and L_2 can be merged to form the composite node C_1 $= \{L_1, L_2\}$. Nodes L_3 and L_4 can also be merged as a composite node $C_2 = \{L_3, L_4\}$. Then, $R_2 \rightarrow \{L_1, L_2, L_3, L_4\}$ is equivalent to $R_2 \rightarrow \{C_1, C_2\}$ which is a V-motif. Thus, $\{L_1, L_2, L_3, L_4\}$ is a cluster (L_1 is a driver).

Another example of determining SI cluster from V-motifs is demonstrated in Supplementary Figures S2d-S2f. In Figure S2d, the new MDS is determined as {4, 6, 13, 14} (blue nodes). Following the procedures introduced in the manuscript, we first find the SI network (Figure S2e). SI clusters are then found by searching for V-motifs in the SI network. The outcome is shown in Figure S2f, which indicates that now the set {14, 1, 5, 7} is the only cluster. Note that {12, 3, 15} is not a cluster since it does not contain a driver node.

Once an SI cluster is identified, it can be removed from the SI network so as to prevent unintended creation of incorrect V-motifs and SI clusters. For example, the SI cluster {4, 6} in Fig. 3c can only be identified after removal of its neighbouring cluster.

Important to note that SI clusters contain no source nodes and in many real situations augmenting to source nodes is impractical. For example, in power systems, generators can be viewed as source nodes (a subset of the driver nodes) and augmenting a new generator to a existing generator is unnecessary.

Supplementary Note 3: Source and sink augmentation can operate in parallel

As we proved earlier, SI (or KI) nodes are the union of nodes in all possible MDSs (or MTSs). We find that for an arbitrary directed network with m MDSs (MDS_1, MDS_2, $\cdots$, MDS_m) and n MTSs (MTS_1, MTS_2, $\cdots$, MTS_n), any pair of MDS_i and MTS_j, has its own unique maximum matching solution, where $i \in (1, 2, \cdots, m)$ and $j \in (1, 2, \cdots, n)$. Hence, adding a source node to a node in MDS_i will not change MTS_j and similarly, adding a sink node to nodes in MTS_j will not affect MDS_i. For example in Supplementary Figure S7, a five node network can be structurally controlled via two possible MDSs: MDS_1={1,2}, MDS_2={1,3}. After implementing maximum matching of its transpose network, we find two possible MTSs: MTS_1={2,5}, MTS_2={3,5}. Then, as shown in Supplementary Figure S7 a, with any combination of an MDS and an MTS, the network remains fully controlled but with different maximum matching solutions. Thus, in effect, one can augment source nodes and sink nodes in parallel.

Supplementary Note 4: Model of random networks

Generating Erdős-Rényi networks

We refer to Erdős-Rényi networks as graphs $\mathrm{G}(N,p)$, which have N nodes and the probability that an edge is present between any pair of nodes is p where $0 \leq p \leq 1$. A graph in $\mathrm{G}(N,p)$ has on average $\binom{N}{2}p$ edges. The out- and in- degree distribution is given by $P_{out,in}(k) = (Np)^k e^{-Np}/k!$ where Np is constant. This distribution is Poisson for large N and the mean-degree is $Np = \langle k_{in} \rangle = \langle k_{out} \rangle = \langle k \rangle /2$.

Generating scale-free networks

The scale-free networks analysed in this paper are generated via the static model as follows [13, 14]. We start from N disconnected nodes indexed by the integers i $(i = 1, 2, \cdots, N)$. Each node i has an out- and an in-weight $w_i^{out,in} = i^{-\alpha_{out,in}}$, where $\alpha_{out,in} = 1/(\gamma_{out,in} - 1)$ and $\gamma_{out,in}$ are exponents of the out- and in-degree distributions. Then, we randomly select two nodes i and j from the set of N nodes, and if they are not connected we connect them. Otherwise, we select a new pair of nodes. The probability of an edge from i to j depends on the out-weight of node i (w_i^{out}) and the in-weight of node j (w_j^{in}). The above process is repeated until the desired number of links are created. Note that $\gamma_{out,in}$ must be greater than or equal to 2. (When $\gamma_{out} = \infty$, we retrieve Erdős-Rényi random networks.) The network typically has a power-law degree distribution. In the large k limit, degree distribution is shown as a function of gamma: $P_{out,in}(k) \sim k^{-\gamma_{out,in}}$.

Supplementary References

1. Milo, R. *et al.* Superfamilies of evolved and designed networks. *Science* **303,** 1538–1542 (2004).
2. Ulanowicz, R., Bondavalli, C, Heymans, J. & Egnotovich, M. Network analysis of trophic dynamics in south florida ecosystem, FY 99: The graminoid ecosystem. *Chesapeake Biological Laboratory, Solomons, MD 20688-0038 USA.* (2000).
3. Ulanowicz, R. C. B. & Egnotovich., M. Network analysis of trophic dynamics in south florida ecosystem, FY 97: The florida bay ecosystem. *Chesapeake Biological Laboratory, Solomons, MD 20688-0038 USA,* 98–123 (1998).
4. Cross, R. L. & Parker, A. *The hidden power of social networks: Understanding how work really gets done in organizations* (Harvard Business Press, 2004).
5. Shen-Orr, S. S., Milo, R., Mangan, S. & Alon, U. Network motifs in the transcriptional regulation network of escherichia coli. *Nature Genetics* **31,** 64–68 (2002).
6. Milo, R. *et al.* Network motifs: simple building blocks of complex networks. *Science* **298,** 824–827 (2002).
7. Shen-Orr, S. S., Milo, R., Mangan, S. & Alon, U. Network motifs in the transcriptional regulation network of escherichia coli. *Nature Genetics* **31,** 64–68 (2002).
8. Bianconi, G., Gulbahce, N. & Motter, A. E. Local structure of directed networks. *Physical Review Letters* **100,** 118701 (2008).
9. Kaiser, M. & Hilgetag, C. C. Nonoptimal component placement, but short processing paths, due to long-distance projections in neural systems. *PLOS Computational Biology* **2,** e95 (2006).
10. Kötter, R. Online retrieval, processing, and visualization of primate connectivity data from the CoCoMac database. *Neuroinformatics* **2,** 127–144 (2004).
11. Choe, B. Y McCormick & Koh, W. Network connectivity analysis on the temporally augmented C. elegans web: A pilot study. *Soc Neurosci Abstr* **30,** 921.9 (2004).
12. Jeong, H., Tombor, B., Albert, R., Oltvai, Z. N. & Barabási, A.-L. The large-scale organization of metabolic networks. *Nature* **407,** 651–654 (2000).
13. Chung, F. & Lu, L. Connected components in random graphs with given expected degree sequences. *Annals of Combinatorics* **6,** 125–145 (2002).
14. Goh, K. I., Kahng, B & Kim, D. Universal behavior of load distribution in scale-free networks. *Physical Review Letters* **87,** 278701 (2001).
